# Poly(lactic-co-glycolic acid) Nanoparticles Encapsulating the Prenylated Flavonoid, Xanthohumol, Protect Corneal Epithelial Cells from Dry Eye Disease-Associated Oxidative Stress

**DOI:** 10.3390/pharmaceutics13091362

**Published:** 2021-08-30

**Authors:** Anita Kirti Ghosh, Rubina Thapa, Harsh Nilesh Hariani, Michael Volyanyuk, Sean David Ogle, Karoline Anne Orloff, Samatha Ankireddy, Karen Lai, Agnė Žiniauskaitė, Evan Benjamin Stubbs, Giedrius Kalesnykas, Jenni Johanna Hakkarainen, Kelly Ann Langert, Simon Kaja

**Affiliations:** 1 Graduate Program in Biochemistry and Molecular Biology, Health Sciences Campus, Loyola University Chicago, Maywood, IL 60153, USA; aghosh3@luc.edu; 2 Visual Neurobiology and Signal Transduction Laboratory, Department of Ophthalmology, Stritch School of Medicine, Loyola University Chicago, Maywood, IL 60153, USA; hhariani@luc.edu (H.N.H.); sogle@luc.edu (S.D.O.); 3 Research Service, Edward Hines Jr. VA Hospital, Hines, IL 60141, USA; evan.stubbs@va.gov; klangert@luc.edu (K.A.L.); 4 Research & Development Division, Experimentica Ltd., 70211 Kuopio, Finland; rubina.thapa@experimentica.com (R.T.); agne@experimentica.com (A.Ž.); jenni@experimentica.com (J.J.H.); 5 Graduate Program in Neuroscience, Health Sciences Campus, Loyola University Chicago, Maywood, IL 60153, USA; mvolyanyuk@luc.edu; 6 Department of Molecular Pharmacology & Neuroscience, Loyola University Chicago, Maywood, IL 60153, USA; kinka@att.net (K.A.O.); srar5b@mail.umkc.edu (S.A.); karen.lai242@gmail.com (K.L.); 7 State Research Institute Centre for Innovative Medicine, Santariskiu 5, LT-08406 Vilnius, Lithuania; 8 Department of Ophthalmology, Stritch School of Medicine, Loyola University Chicago, Maywood, IL 60153, USA; 9 Research & Development Division, UAB Experimentica, LT-10223 Vilnius, Lithuania; gk@experimentica.com; 10 North Texas Eye Research Institute, University of North Texas Health Science Center at Fort Worth, Fort Worth, TX 76107, USA

**Keywords:** ocular surface disease, dry eye disease, antioxidant, xanthohumol, drug delivery, PLGA nanoparticles

## Abstract

Oxidative stress is a known contributor to the progression of dry eye disease pathophysiology, and previous studies have shown that antioxidant intervention is a promising therapeutic approach to reduce the disease burden and slow disease progression. In this study, we evaluated the pharmacological efficacy of the naturally occurring prenylated chalconoid, xanthohumol, in preclinical models for dry eye disease. Xanthohumol acts by promoting the transcription of phase II antioxidant enzymes. In this study, xanthohumol prevented *tert*-butyl hydroperoxide-induced loss of cell viability in human corneal epithelial (HCE-T) cells in a dose-dependent manner and resulted in a significant increase in expression of the transcription factor nuclear factor erythroid 2-related factor 2 (Nrf2), the master regulator of phase II endogenous antioxidant enzymes. Xanthohumol-encapsulating poly(lactic-co-glycolic acid) nanoparticles (PLGA NP) were cytoprotective against oxidative stress in vitro, and significantly reduced ocular surface damage and oxidative stress-associated DNA damage in corneal epithelial cells in the mouse desiccating stress/scopolamine model for dry eye disease in vivo. PLGA NP represent a safe and efficacious drug delivery vehicle for hydrophobic small molecules to the ocular surface. Optimization of NP-based antioxidant formulations with the goal to minimize instillation frequency may represent future therapeutic options for dry eye disease and related ocular surface disease.

## 1. Introduction

Dry eye disease is an umbrella term describing various subtypes of the disease. Dry eye disease poses a substantial burden on the affected individual and society as a whole. Existing pharmacologic management for dry eye disease targets T cell-mediated inflammatory pathways and in the United States consists of ophthalmic formulations of cyclosporine (Restasis^®^ or Cequa™) or the lymphocyte function-associated antigen 1 inhibitor, lifitegrast (Xiidra^®^). Both agents are associated with limited efficacy and adverse effects in up to 25% of patients [1,2,3], highlighting an urgent unmet clinical need for novel efficacious and well-tolerated therapeutics.

Previous studies have implicated the generation of reactive oxygen species (ROS) and the ensuing elevated levels of cellular oxidative stress as a key contributor to the pathophysiology of dry eye disease (reviewed in [4]). Specifically, elevated levels of oxidative stress have been identified in patients with dry eye disease [5,6], while hyperosmolar conditions cause oxidative stress in cultured corneal epithelial cells [7]. We have recently shown significant oxidative DNA damage in the corneal epithelium of mice exposed to dry eye-inducing conditions of desiccating environment with scopolamine [8]. Similarly, lacrimal gland dysfunction as a result of mitochondrial oxidative stress produces an ocular phenotype reminiscent of dry eye disease in mice [9,10].

Notably, a mitochondrially-targeted antioxidant, SkQ1 (Visomitin), exerts anti-inflammatory effects in human conjunctival epithelial cells in vitro [11], and has shown therapeutic benefit in US Phase 2 clinical trials following approval in Russia in 2011 [12], providing proof-of-concept evidence supporting the development of therapeutic approaches using antioxidants to treat dry eye disease.

Major challenges associated with dry eye disease management are low patient satisfaction and poor compliance with dosing regimens [13]. Therefore, one important drug development consideration for topical ophthalmic formulations is to enhance ocular surface retention times that minimize the number of instillations.

In this study, we evaluated the anti-oxidative and anti-inflammatory properties of xanthohumol in preclinical models for dry eye disease. Xanthohumol is a naturally occurring prenylated chalconoid that is abundantly present in *Humulus lupulus*, the hops plant. Xanthohumol promotes the transcription of phase II antioxidant enzymes [14], by stimulating the dissociation of Kelch-like ECH-associated protein 1 (Keap1) from Nuclear factor erythroid 2-related factor 2 (Nrf2), the master regulator of the endogenous antioxidant response. Keap1 is the main negative regulator of Nrf2, targeting it for ubiquitylation and degradation. The dissociation of Keap1 from Nrf2 results in nuclear translocation of Nrf2 and subsequent activation of gene expression driven by the antioxidant response element. In addition, xanthohumol exhibits direct ROS scavenging activity due to its chalconoid structure [15].

Xanthohumol was selected based on the rationale that exploiting its dual mechanism of boosting the endogenous antioxidant response by relieving Keap1 suppression of Nrf2 translocation and direct ROS scavenging may be advantageous over antioxidants with only direct ROS scavenging activity.

The objectives of this study were to determine the cytoprotective effects of xanthohumol in human corneal epithelial cells in vitro, and in the mouse desiccating stress/scopolamine model for dry eye disease in vivo, using both non-formulated and poly(lactic-co-glycolic acid) nanoparticle (PLGA NP)-encapsulating xanthohumol.

## 2. Materials and Methods

### 2.1. Test Articles, Antibodies and Chemicals

Xanthohumol was purchased from Cayman Chemicals (Ann Arbor, MI, USA) and dissolved in dimethyl sulfoxide at a concentration of 100 µM (Millipore Sigma, St. Louis, MO, USA) for in vitro experiments. Cyclosporine A for transporter assays was USP grade (≤99% purity) from Cayman Chemical Company (Ann Arbor, MI, USA). Ophthalmic cyclosporine emulsion was pharmaceutical grade, Restasis^®^ (0.05% cyclosporine; Allergan Plc., Irvine, CA, USA).

The following antibodies were used for immunoblotting experiments: mouse anti-NFE2L2 (Nrf2; VMA00224; BioRad Laboratories Inc., Hercules, CA, USA; 1:1000 dilution). Glyceraldehyde-3-phosphate dehydrogenase (GAPDH) was used as endogenous control (rabbit anti-GAPDH; sc-25778; Santa Cruz Biotechnology, Dallas, TX, USA; 1:2000 dilution). Secondary antibodies were horseradish peroxidase-conjugated and obtained from GE Healthcare (Chicago, IL, USA). Anti-8-OHdG antibody (clone N45.1, 1:200 dilution, Japan Institute for the Control of Aging, NIKKEN SEIL Co., Ltd., Shizuoka, Japan) was used for 8-OHdG staining on corneal sections.

Unless otherwise specified, analytical grade reagents were obtained from Millipore Sigma (St. Louis, MO, USA).

### 2.2. Cell Culture

Human corneal epithelial cells (HCE-T; RIKEN BioResource Research Center, Tsukuba, Japan; [16]) were cultured as described previously [8,17,18,19]. Specifically, HCE-T cells were maintained in standard tissue culture flasks (Techno Plastic Products, MidSci, St. Louis, MO, USA) in a humidified atmosphere supplemented with 5% CO_2_ at 37 °C. Growth medium was comprised of DMEM/F12 (1:1) (Thermo Fisher Scientific, Waltham, MA, USA) with 5% fetal bovine serum (Gemini Bio Products, West Sacramento, CA, USA), 0.5% dimethyl sulfoxide, 5 µg/mL insulin (both from Millipore Sigma), 10 ng/mL human recombinant epidermal growth factor, and 100 U/mL penicillin–100 mg/mL streptomycin (both from Thermo Fisher Scientific, Waltham, MA, USA). Cultures of passages 79 to 95, were used for experiments.

### 2.3. Cell Viability Assays

To determine the cytoprotective effects of xanthohumol against chemically-induced oxidative stress, we conducted 3-(4,5-dimethylthiazol-2-yl)-2,5-diphenyltetrazolium bromide (MTT) uptake and lactate dehydrogenase (LDH) release assays, essentially as previously described [8]. Briefly, HCE-T cells were seeded in 96-well plates (Techno Plastic Products, MidSci, St. Louis, MO, USA) at a density of 10,000 cells/well. Once confluent, cells were pre-treated with xanthohumol (0.1, 0.5, 1 or 5 µM) for 20 h, and subsequently exposed to a range of *tert*-butyl hydroperoxide (*t*BHP) concentrations (5–500 µM) for 6 h. MTT and LDH assays were performed immediately following tBHP exposure.

For LDH assay, 50 µL of the supernatants were transferred to a new 96-well plate (Nunc™, Thermo Fisher Scientific, Waltham, MA, USA). An equal volume of LDH assay buffer (containing 2 mM iodonitrotetrazolium chloride, 3.2 mM β-nicotinamide adenine dinucleotide sodium salt, 160 mM lithium lactate, 7.5 µM 1-methoxyphenazine methosulfate in 0.2 M Tris-HCl buffer, pH 8.2) was added to the wells. Supernatants with assay buffer were incubated at room temperature in the dark for 1 h. The reaction was stopped by addition of 50 µL of 1 M acetic acid. Absorbance was measured at λ = 490 nm using a Cytation 5 imaging plate reader (Biotek, Winooski, VT, USA).

For MTT assay, a 12 mM stock solution of MTT was prepared in Hank’s Balanced Salt Solution with calcium and magnesium (Lonza, Walkersville, MD, USA) that was stored frozen until use. On the day of experiments, a 1.2 mM working stock was prepared by diluting the stock solution in Hank’s Balanced Salt Solution with calcium and magnesium supplemented with 10 mM 4-(2-hydroxyethyl) piperazine-1-ethanesulfonic acid and the pH adjusted to 7.3. Media were aspirated and cells were incubated with 100 µL MTT working solution for 2 h in a 37 °C oven. MTT was aspirated from the cells and cells were lysed with 100 µL dimethyl sulfoxide while gently shaking. Absorbance was measured at λ = 570 nm using a Cytation 5 imaging plate reader (Biotek, Winooski, VT, USA).

For both MTT and LDH assays, each experiment (n) is derived from 4–8 technical replicates per condition; n numbers in the text and figure legends represent the number of biological replicates. Data were exported to Microsoft Excel (Microsoft Corporation, Redmond, WA, USA), normalized to the baseline control condition (no tBHP) and expressed as fold-change. Cell viability data were fitted in Prism 9.0 software (GraphPad, Inc., La Jolla, CA) by non-linear regression using a four-parameter logistic equation with variable Hill slope, as described previously [19]. To determine the half-maximal effect sizes (EC_50_ and IC_50_), non-linear regression was performed separately for each biological replicate, consisting of 4–8 technical replicates.

### 2.4. Quantitative Immunoblotting

Immunoblotting on HCE-T cell lysates was performed as described previously [17]. Briefly, media were aspirated, and cells were rinsed in phosphate-buffered saline (PBS). Cells were scraped in ice cold PBS, samples centrifuged at 800 × *g* for 5 min, supernatants aspirated, and pellets lysed in CytoBuster^TM^ lysis reagent (Millipore Sigma, St. Louis, MO, USA) containing protease inhibitor cocktail (Thermo Fisher Scientific, Waltham, MA, USA). Samples were triturated with a 31-gauge insulin syringe and centrifuged at 16,000× *g* for 10 min. Lowry assays [20] were performed on the supernatants to determine protein sample concentrations.

Samples were diluted to the same protein concentration using CytoBuster^TM^ lysis buffer, supplemented with sodium dodecyl sulfate loading buffer (Morganville Scientific, Morganville, NJ, USA) and stored at −80 °C until use. Immediately prior to electrophoresis, samples were denatured in a heat block at 85 °C for 5 min. Gels (pre-cast 4–12% NuPage^®^ Bis/Tris; Thermo Fisher Scientific, Waltham, MA, USA) were loaded with 10 µg of each protein sample, and electrophoresed at 150 V for 75 min. Wet transfer of proteins from gel to a nitrocellulose membrane with 0.1 µM pore size (Amersham Protran, GE Healthcare, Chicago, IL, USA) was performed at 100 V for 90 min in Pierce^TM^ Methanol-free Western Blot Transfer Buffer (Thermo Fisher Scientific, Waltham, MA, USA). The membrane was blocked in 5% non-fat milk in PBS supplemented with 0.2 % Tween-20 (PBS-T), then incubated with primary antibody (anti-Nrf2 and anti-GAPHDH, as described above) in 2.5% milk in PBS-T overnight at 4 °C on a shaker. Membrane was washed three times for 10 min each in PBS-T, then incubated with horseradish peroxidase-linked secondary antibody in 2.5% milk in PBS-T for 1 h at room temperature. Chemiluminescence was performed using Luminata Forte^®^ (Millipore Sigma, St. Louis, MO, USA) and images acquired using a ChemiDoc™ XRS+ (Bio-Rad Laboratories, Hercules, CA, USA). Relative protein expression was quantified by densitometry using Image Lab software (Bio-Rad Laboratories, Hercules, CA, USA) and normalized to endogenous control GAPDH and to the control or vehicle condition.

### 2.5. Generation and Characterization of PLGA NP

Empty PLGA NP were prepared using an oil-in-water single emulsion technique, essentially as described previously [21]. Briefly, 50 mg of PLGA (85:15; Durect Corp., Birmingham, AL, USA) were dissolved in 1 mL dichloromethane and slowly added to ice-cold polyvinyl alcohol (1% *w*/*v*, 10 mL), while vigorously vortexing. The resultant suspension was emulsified by probe sonication and diluted with 100 mL ice-cold polyvinyl alcohol. The organic solvent was allowed to evaporate with constant stirring for 3 h at 23 °C and the resulting PLGA NP were isolated by centrifugation (25,000× *g* for 20 min at 4 °C) and washed three times with deionized water.

Xanthohumol-encapsulating PLGA NP were synthesized as above, with xanthohumol (5 mg) added to the initial organic phase (PLGA in dichloromethane). Both empty and xanthohumol-encapsulating PLGA NP were resuspended in sucrose (10 mL of 5 mg/mL sucrose in deionized water) and lyophilized. PLGA NP were stored at −80 °C until use.

PLGA NP were characterized morphologically by transmission electron microscopy (TEM). To this end, PLGA NP were suspended at a concentration of 10 mg/mL in physiological saline. Carbon-coated 200 mesh copper grids (Electron Microscopy Sciences, Hatfield, PA, USA) pre-treated with 0.002% Alcian blue in 0.03% acetic acid were floated on top of 30 µL drops of NP suspensions (30 min, room temperature). After washing with diH_2_O, the samples were negatively stained by floating the grid on 50 µL drop of sterile-filtered uranyl acetate (pH = 7, 5 min, room temperature). Samples were dried for 12 h in a grid storage box before imaging with a Phillips CM120 transmission electron microscope (TSS Microscopy, Beaverton, OR, USA) equipped with a BioSprint digital camera (Advanced Microscopy Techniques, Woburn, MA, USA).

PLGA NP properties were determined by dynamic light scattering using a ZetaSizer analyzer (Malvern Pananalytical Inc., Westborough, MA, USA).

### 2.6. Bioanalytical High-Performance Liquid Chromatography (HPLC) Method for Xanthohumol Detection

Xanthohumol was quantified by high-performance liquid chromatography (HPLC) using a LaChrom Elite (Hitachi High-Tech Analytical Science, Westford, MA, USA) analytical system equipped with a model L-2130 pumping station, an autosampler/autoinjector (model L-2200), a column oven system (model L-2300), a UV-Vis detector (model L-2420) and subsequently analyzed using EZChrom Elite analytical software. Xanthohumol encapsulated PLGA nanoparticles were dissolved in dimethyl sulfoxide (4.5 mg/mL) and the liberated xanthohumol resolved on a HiChrom Ultrasphere 5 µm C18 reverse-phase column (4.6 mm × 25 cm) at a flow rate 1.0 mL min^−1^. Xanthohumol was eluted from the column using a non-linear gradient of acetonitrile balanced with 0.05 N formic acid over a total run time of 25 min at 23 °C as follows: 20–50% acetonitrile (0–3 min); 50–70% acetonitrile (3–6 min); 70–100% acetonitrile (6–15 min); 100% acetonitrile (15–20 min); 100–20% acetonitrile (20–25 min). When analyzed at 370 nm, xanthohumol eluted as a single peak with a retention time of 11.6 min. The eluted xanthohumol was identified by comparison of the retention time and the UV spectra with that obtained using pure standard (Cayman Chemicals, Ann Arbor, MI, USA) injected under identical chromatographic conditions. The quantity of liberated xanthohumol was determined using a 9-point linear (r^2^ = 0.99; LOD = 1 pmol) calibration curve ranging from 0–10 nmols xanthohumol standard prepared in dimethyl sulfoxide.

### 2.7. Desiccating Stress/Scopolamine Model for Experimental Dry Eye Disease

All animals were treated in accordance with the ARVO Statement for the Use of Animals in Ophthalmic and Vision Research and the European Commission Directive 86/609/EEC for animal experiments, using protocols approved and monitored by the Animal Experiment Board of Finland (protocol number ESAVI-10756-2020, approved 5/19/2020). C57BL/6JRj mice were purchased from Janvier Labs (Le Genest-Sainte-Isle, France). Mice were housed at a constant temperature (22 ± 1 °C) and in a light-controlled environment (lights on from 7 a.m. to 7 p.m.) with *ad libitum* access to food and water. Male mice (9 weeks of age) were used for experiments.

Dry eye disease-like pathology was induced by exposure to a combination of desiccating stress in SiccaSystem^®^ cages (K&P Scientific LLC, Forest Park, IL, USA) and transdermal administration of scopolamine (Scopoderm^®^; Glaxo Smith Kline, Middlesex, UK), as described previously [8,22]. Briefly, each 1 mg scopolamine patch was punched into 14 smaller pieces, each containing approximately 70 µg of scopolamine. Each mouse was administered a transdermal patch in each ear (total 140 µg). The presence of patches was checked daily, and patches were replaced every 72 h.

The SiccaSystem^®^ cages allow animals to be exposed to a desiccating environment of 5–15% humidity with 15 L/min airflow. In this study, mice were exposed to desiccating stress/scopolamine for a total of 26 days; test articles (empty PLGA NP, xanthohumol-encapsulating PLGA NP, or cyclosporine) were administered twice daily (8 a.m. and 5 p.m.) by topical instillation (10 µL) into both eyes starting on day 16 for a period of ten days.

Tear volume was quantified using phenol red-coated threads (ZoneQuick^®^; FCI Ophthalmics, Pembroke, MA, USA) [8,22]. The thread was placed in the lateral canthus for 30 s, and a blinded investigator measured the wet length of the thread (in mm) using a ruler. Tear volumes were measured on day 0 (baseline), 15, and 26 (before euthanasia) of the study.

Corneal fluorescein staining measurements were performed on day 15 and on day 26, essentially as described previously [8,22]. Briefly, 2 µL of 0.2 % liquid sodium fluorescein was pipetted into to the conjunctival sac in both eyes of each animal. Fluorescence retained on the ocular surface was imaged using a fluorescent microscope (Leica Microsystems, Buffalo Grove, IL, USA).

Animals were randomized and assigned to treatment groups based on the corneal fluorescein score on day 15, such that the median and interquartile range of fluorescein scores were similar between groups at the onset of treatment (day 16). Scoring of fluorescent images was performed by two blinded investigators based on established criteria [16]. To determine the pharmacologic efficacy of xanthohumol-encapsulating PLGA NP, fluorescence intensity was quantified from images using Fiji software [23].

### 2.8. 8-Hydroxy-2′-deoxyguanosine (8-OHdG) Staining

On study day 26, after corneal fluorescein imaging, mice were euthanized by thoracotomy following intraperitoneal administration of 75 mg/kg ketamine and 1 mg/kg xylazine. 8-OHDG staining was performed as described in detail previously [8]. Briefly, excised corneas were fixed in 4% paraformaldehyde in PBS and cryoprotected in serial sucrose solution (10%, 20%, 30% *w/v* in PBS) and cryosections of cornea were labeled with an anti-8-OHdG antibody (clone N45.1, 1:200 dilution, Japan Institute for the Control of Aging, NIKKEN SEIL Co., Ltd., Shizuoka, Japan). 8-OHdG immunoreactivity was quantified by measuring density of nuclei in the corneal epithelial layer. Density of eight randomly-selected nuclei in three regions of approx. 100 μm^2^ was measured using Fiji software [23]. Values were averaged to obtain the mean for that eye.

### 2.9. Data Analysis and Statistics

All data were analyzed with the investigator blinded for treatment group. Data are presented as mean ± standard error of mean (SEM) or as median ± interquartile range or 25th/75th percentile. Data were analyzed using unpaired Student’s *t*-test (8-OHdG staining), Kruskal-Wallis ANOVA (effect sizes of tear volume measurements) or two-way ANOVA. Homoscedasticity tests were performed by computing the nonparametric correlation between the absolute values of the residuals and the Y value of the curve. Differences between groups on homoscedastic data sets were subsequently determined using Šidák’s multiple comparisons test as appropriate. When computing a repeated measures two-way ANOVA (i.e., for tear volume measurements), differences between groups were determined by Tukey’s multiple comparisons test. Differences were considered statistically significant at the *p* < 0.05 level. Statistical analysis was performed using GraphPad Prism 9 software (GraphPad Software, San Diego, CA, USA).

## 3. Results

### 3.1. Xanthohumol Exerts Cytoprotective Effects against Chemically-Induced Oxidative Stress in HCE-T Cells

**Figure 1:**
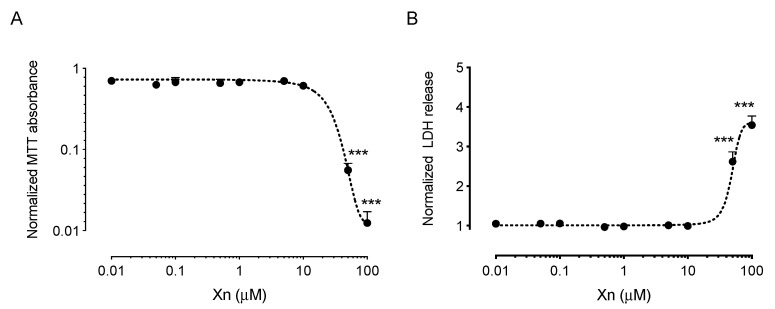
Cytotoxicity of xanthohumol in HCE-T cells. (**A**) Xanthohumol resulted in dose-dependent cytotoxicity in HCE-T cells as shown by MTT assay. Concentrations up to 10 µM did not have a statistically significant effect on cell proliferation and survival. Concentrations of 50 µM and 100 µM resulted in a reduction of MTT absorbance by 94.4 ± 1.2% and 98.8 ± 0.5%, respectively (*n* = 3); (**B**) Similarly, xanthohumol concentrations greater than 10 µM resulted in a statistically significant increase in LDH release (2.6 ± 0.2-fold at 50 µM and 3.5 ± 0.2-fold at 100 µM; *n* = 3). Data are shown as mean ± SEM. *** *p* < 0.001. Xn = xanthohumol.

In order to determine the cytotoxicity of xanthohumol, human corneal epithelial (HCE-T) cells were exposed to a concentration range of xanthohumol (10 nM–100 µM) and incubated for 48 h. Dimethyl sulfoxide vehicle was kept constant at 0.1% weight/volume for all xanthohumol concentrations. Cell survival and proliferation were assessed by 3-(4,5-dimethylthiazol-2-yl)-2,5-diphenyltetrazolium bromide (MTT) uptake and lactate dehydrogenase (LDH) release assays. Xanthohumol concentrations up to 10 µM had no effect on MTT absorbance (*p* < 0.001, *n* = 3; Figure 1A) or LDH release (*p* < 0.001, *n* = 3; Figure 1B), when compared to the dimethyl sulfoxide vehicle condition. Higher concentrations of xanthohumol exerted dose-dependent cytotoxicity, resulting in almost complete loss of cell viability at 100 µM (*p* < 0.001, *n* = 3; Figure 1).

**Figure 2:**
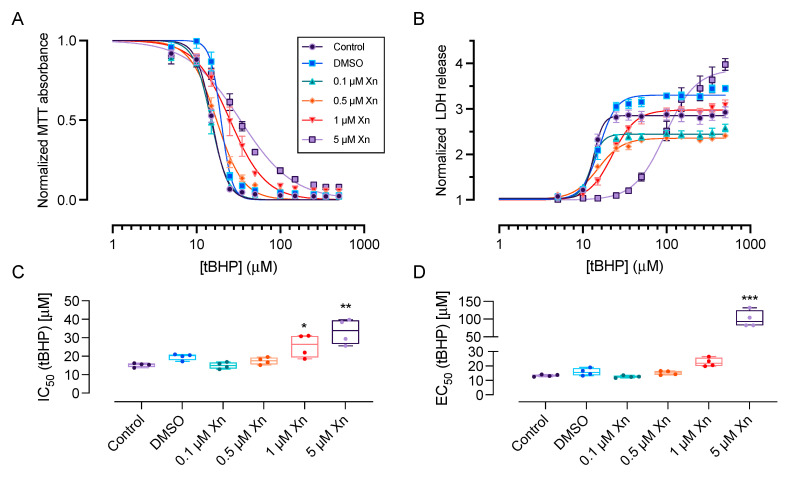
Xanthohumol exerts dose-dependent cytoprotective effects against tBHP-induced oxidative stress in HCE-T cells. (**A**) Xanthohumol (0.1–5 µM) caused a right-shift of dose-response curves for tBHP in the MTT assay, suggestive of cytoprotection. Data were fitted using a four-parameter dose response curve. (**B**) Similarly, xanthohumol (0.1–5 µM) resulted in a right-shift of the LDH response. Data were normalized to the no tBHP control for each condition. (**C**) Quantification of the IC_50_ values from the MTT assay revealed an approximately 2.2-fold increase in the presence of 5 µM xanthohumol (15.2 ± 0.5 µM vs. 33.3 ± 3.4 µM, *p* < 0.01, *n* = 4). (**D**) In the LDH assay, xanthohumol (5 µM) increased the EC_50_ for tBHP from 13.4 ± 0.4 µM to 100.0 ± 11.7 µM (*p* < 0.001; *n* = 4). Data were analyzed by two-way ANOVA with Šídák’s multiple comparisons test and are shown as mean ± SEM. * *p* < 0.05, ** *p* < 0.01, *** *p* < 0.001. Xn = xanthohumol.

Based on the results from cytotoxicity assays, we selected four sublethal concentrations of xanthohumol (0.1 µM, 0.5 µM, 1 µM and 5 µM) to determine the cytoprotective and antioxidant effects against exogenously-applied *tert*-butyl hydroperoxide (*t*BHP)-induced oxidative stress. HCE-T cells were exposed to xanthohumol for 20 h, and subsequently exposed to a concentration range of tBHP (5–500 µM) for 6 h prior to performing MTT and LDH assays (Figure 2). For these studies, dimethyl sulfoxide vehicle was used at a concentration of 0.005% weight/volume.

Xanthohumol resulted in a dose-dependent protection against oxidative stress, as evident by a right-shift in the IC_50_ curves for tBHP in the MTT assay (Figure 2A). Similarly, xanthohumol caused a right-shift in the EC_50_ curves for tBHP in the LDH assay (Figure 2B). Specifically, the IC_50_ for tBHP in the MTT assay was 15.2 ± 0.5 µM in the control condition. 1 µM and 5 µM xanthohumol resulted in a statistically significant increase in the IC_50_ values for tBHP to 25.6 ± 3.2 µM (*p* < 0.05, *n* = 4) and 33.3 ± 3.4 µM (*p* < 0.01, *n* = 4; Figure 2C), respectively.

Similarly, xanthohumol (5 µM) increased the EC_50_ value for tBHP in the LDH assay from 13.4 ± 0.4 µM to 100.0 ± 11.7 µM (*p* < 0.001; *n* = 4; Figure 2D).

Dimethyl sulfoxide vehicle alone (0.005% weight/volume) did not result in any statistically significant reduction in cell viability in response to tBHP insult (Figure 2).

### 3.2. Xanthohumol Elicits Significant Increase in Nrf2 Protein Levels in Human Corneal Epithelial Cells

Xanthohumol is a well-known activator of the endogenous antioxidant system that acts by stimulating the dissociation of Keap1 from Nrf2. In order to demonstrate the ability of xanthohumol to elicit this effect in corneal epithelial cells, we performed a time course analysis of Nrf2 protein levels after exposure to xanthohumol in HCE-T cells.

**Figure 3:**
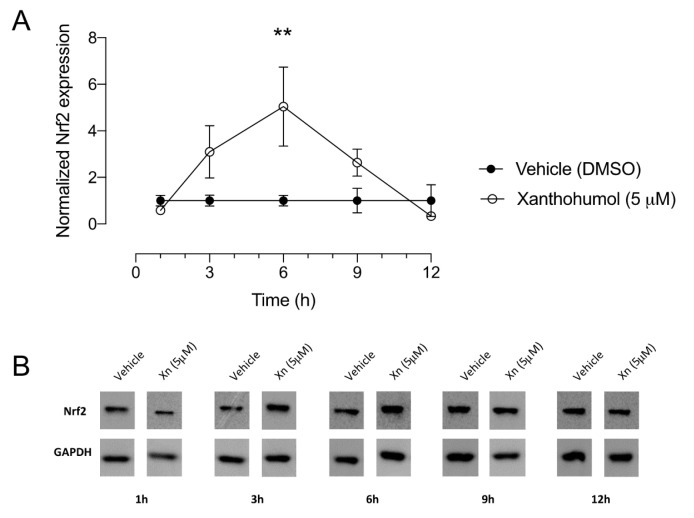
Xanthohumol increases protein levels of Nrf2 in human corneal epithelial cells. (**A**) Quantification of immunoblots revealed a statistically significant 5-fold increase of Nrf2 in xanthohumol-treated cells compared with vehicle after 6 h incubation. Data were analyzed by two-way ANOVA with Šídák’s multiple comparisons test and are shown as mean ± SEM from three separate experiments. ** *p* < 0.01. (**B**) Representative examples of Nrf2 immunoblots from xanthohumol-versus vehicle-treated cell lysates are shown. GAPDH was used as endogenous control. Xn = xanthohumol.

Nrf2 protein levels peaked after 6 h of xanthohumol (5 µM) and were 5.0 ± 1.7-fold higher than in vehicle-treated cells (1.0 ± 0.2; *n* = 3, *p* < 0.01; Figure 3).

Together with results from the cell viability assays presented in Figure 2, our data suggest that xanthohumol can exert antioxidant effects in human corneal epithelial cells.

### 3.3. Xanthohumol-Encapsulating PLGA NP Are Cytoprotective against Oxidative Stress in HCE-T Cells

We next generated xanthohumol-encapsulating PLGA NP using an 85:15 ratio of poly-lactic and poly-glycolic acid, based on previously established release parameters [21]. Nanoparticle formulations were resuspended in saline and their properties analyzed by Dynamic Light Scattering using a ZetaSizer (Malvern Pananalytical Inc., Westborough, MA, USA). Encapsulation efficiency of xanthohumol was 13.1 ± 0.06%, as determined by bioanalytical detection of xanthohumol by HPLC against a standard curve of purified xanthohumol. Xanthohumnol eluted as a single peak at 11.6 min retention time.

**Figure 4:**
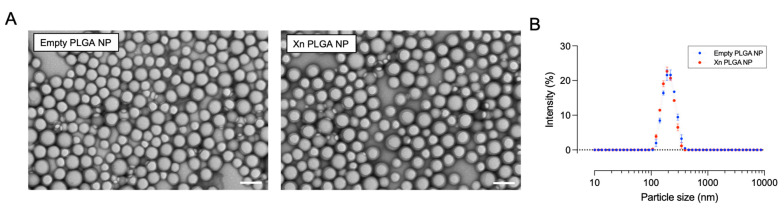
Morphology and size distribution of PLGA NP. (**A**) Representative transmission electron micrographs (17,000× magnification) depicting Empty PLGA NP and Xn-encapsulating PLGA NP. Scale bars: 200 nm. (**B**) Empty and xanthohumol-encapsulating PLGA NP have similar sizes and unimodal size distribution. Data are mean ± s.e.m. from three different PLGA NP preparations, each tested in triplicate. Xn = xanthohumol.

Empty and xanthohumol-encapsulating PLGA NP were similar in size and size distribution, averaging ~200 nm (Figure 4; Table 1). Similarly, the polydispersity index was below 0.05 for both PLGA NP formulations, suggesting a unimodal size distribution and absence of aggregation (Table 1). The surface charge of PLGA NP was negative, in line with previous observations [21] (Table 1).

To assess the cytotoxicity of PLGA NP and release of xanthohumol, we performed cell viability assays in HCE-T cells analogous to the experiments described above. HCE-T cells were seeded in 96 well plates and incubated with increasing amounts of empty and xanthohumol-encapsulating PLGA NP for 48 h. The concentration of xanthohumol represents the total amount of xanthohumol present in the NP applied to the cells. In the control condition, cells were exposed to an equivalent amount (milligrams) of empty PLGA NP.

**Figure 5:**
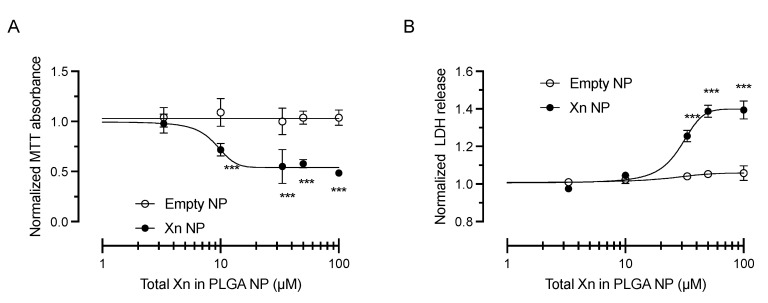
Cytotoxicity of xanthohumol-encapsulating PLGA NP in HCE-T cells. (**A**) Xanthohumol-encapsulating PLGA NP resulted in dose-dependent cytotoxicity in HCE-T cells as shown by MTT assay. Concentrations ≥10 µM exerted a statistically significant effect on cell proliferation and survival after 48 h incubation when compared to matching amount of empty PLGA NP (*n* = 3–5, *p* < 0.001); (**B**) Similarly, xanthohumol concentrations of >10 µM resulted in a statistically significant increase of LDH release (*n* = 3–5, *p* < 0.001). Data were analyzed by two-way ANOVA with Šídák’s multiple comparisons test and are shown as mean ± SEM. *** *p* < 0.001. Xn = xanthohumol.

Increasing concentrations of xanthohumol-encapsulating PLGA NP exerted a dose-dependent toxicity as evident by a decrease in MTT absorbance (*n* = 3–5, *p* < 0.001; Figure 5A) and a concomitant increase in LDH release (*n* = 3–5; *p* < 0.001; Figure 5B). In contrast, increasing amounts (matching the NP amount of each Xn NP dose) of empty PLGA NP did not exert any cytotoxicity (Figure 5A,B). Differences were statistically evaluated by two-way ANOVA with Šídák’s multiple comparisons test, indicating that concentration of 10 µM or higher resulted in statistically significant cytotoxicity in HCE-T cells.

**Figure 6:**
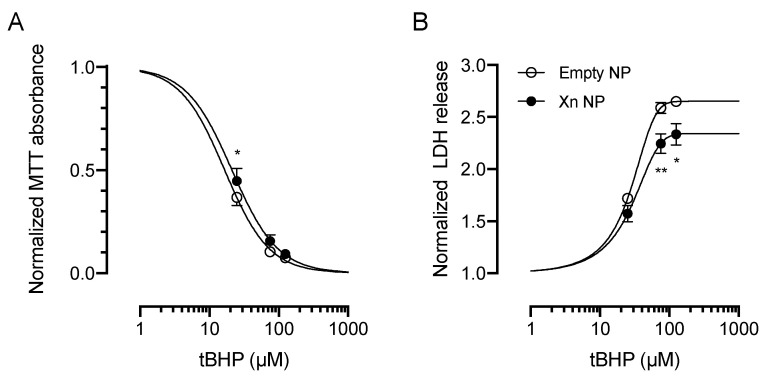
Xanthohumol-encapsulating PLGA NP showed cytoprotection against tBHP-induced oxidative stress in HCE-T cells. (**A**) Xanthohumol-encapsulating PLGA NP (5 µM) caused a right-shift of the dose-response curves for tBHP in the MTT assay, shifting the IC_50_ value by 4.6 µM (*n* = 3, *p* < 0.05). (**B**) Similarly, xanthohumol-encapsulating PLGA NP (5 µM) resulted in a right-shift of the LDH response, increasing the EC_50_ value by 4.5 µM (*n* = 3, *p* < 0.01). Data are shown as mean ± SEM and were fitted using a four-parameter dose response curve. Data were analyzed by two-way ANOVA followed by Šídák’s multiple comparisons test. * *p* < 0.05, ** *p* < 0.01. Xn NP = xanthohumol-encapsulating PLGA NP.

Next, we tested the ability of xanthohumol-encapsulating NP to protect HCE-T cells from exogenously-applied oxidative stress. We incubated HCE-T cells with either empty or xanthohumol-encapsulating (5 µM) PLGA NP for 20 h, prior to exposing HCE-T cells to a dose-range of tBHP (25–125 µM) for 5 h. Xanthohumol-encapsulating PLGA NP caused a statistically significant shift in the dose-response to tBHP (*n* = 3, *p* < 0.01; Figure 6A), with IC_50_ values for tBHP increasing from 16.6 µM (interquartile range: 14.1–18.9 µM) to 21.2 µM (interquartile range: 17.9 µM to 24.1 µM. Similarly, EC_50_ for tBHP values derived from the LDH release assay increased significantly from 17.9 µM to 22.4 µM (*n* = 3, *p* < 0.01; Figure 6B).

Based on these data, we have identified a safe dose of xanthohumol-encapsulating PLGA NP in HCE-T cells and confirm that xanthohumol delivered via PLGA NP can exert antioxidative effects in human corneal epithelial cells. In the next set of experiments, we tested the efficacy of xanthohumol-encapsulating PLGA NP in a preclinical dry eye disease model.

### 3.4. Xanthohumol-Encapsulating PLGA NP Reverse Ocular Surface Damage in the Desiccating Stress/Scopolamine Model for Dry Eye Disease

We used the mouse desiccating stress/scopolamine model to test the efficacy of xanthohumol-encapsulating PLGA NP. Mice were exposed to SiccaSystem^®^ cages for a period of 15 days without intervention. Subsequently, mice were treated twice daily (8 a.m. and 5 p.m.) by topical instillation of either empty PLGA NP, xanthohumol-encapsulating PLGA NP or cyclosporine. In this study, we did not include a separate vehicle control group, as we have previously determined that empty PLGA NP do not exert any cytoprotective effects compared with 0.9% saline solution (data not shown).

**Figure 7:**
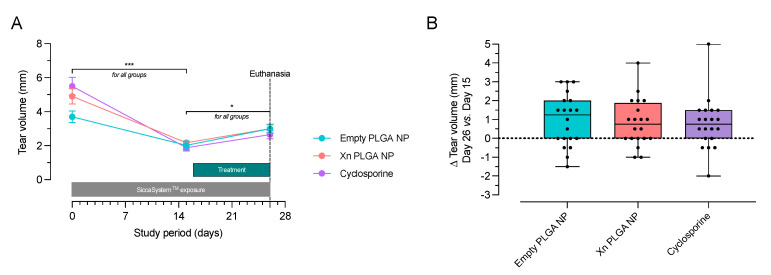
Xanthohumol PLGA NP do not affect tear volumes. (**A**) Tear volumes decreased significantly as a result of the exposure to the desiccating stress/scopolamine environment (*n* = 20, *** *p* < 0.001 for all groups); all treatments significantly increased tear volumes (*n* = 20, * *p* ≤ 0.05 for all groups), as determined by two-way ANOVA. Data are shown as mean ± SEM. (**B**) Comparison of effect sizes determined by calculating the difference in tear volume between day 25 and day 16 did not show any statistically significant differences (*n* = 20, *p* = 0.86, Kruskal–Wallis ANOVA). Data are shown as box and whisker plot, indicating the median (line), with the box extending from the 25th to 75th percentiles. Whiskers represent the range, while filled circles are individual data points from a single eye. Xn = xanthohumol.

First, we quantified tear volumes, at baseline, before start of topical treatments on day 15 and at the end of the study on day 26. We observed a statistically significant reduction of tear volumes on Day 15 suggestive of successful induction of dry eye disease pathology (from 4.7 ± 0.3 mm to 2.0 ± 0.1 mm, n = 60 eyes, *p* < 0.001). Two-way ANOVA analysis revealed a statistically significant effect of time (*p* < 0.001), but not treatment (*p* = 0.29), and tear volumes showed a similar statistically significant increase of tear volumes from day 15 to day 26 (*p* < 0.05 for all treatment groups using Tukey’s multiple comparisons test; Figure 7A). Effect sizes for each treatment, determined by calculating the difference between tear volume and day 15 and day 26, did also not differ between treatment groups (Kruskal–Wallis ANOVA, *p* = 0.86; Figure 7B).

**Figure 8:**
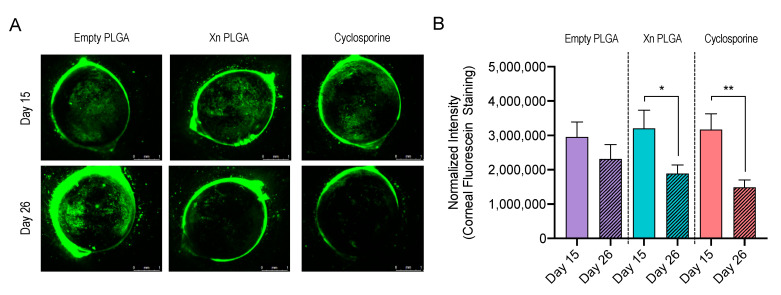
Xanthohumol-encapsulating PLGA NP reduce corneal fluorescein staining in the mouse desiccating stress/scopolamine model for dry eye disease. (**A**) Representative examples of corneal fluorescein staining from day 15 and day 26. Scale bar: 1 mm. (**B**) Quantification revealed a statistically significant reduction of corneal fluorescein staining by xanthohumol-encapsulating PLGA NP and cyclosporine, while empty PLGA NP had no significant effect on fluorescein staining (*n* = 18–20 eyes per group), as determined by Mann–Whitney test. * *p* < 0.05, ** *p* < 0.01. Data are shown as mean ± SEM. Xn = xanthohumol.

In order to determine possible effects on corneal damage, we performed corneal fluorescein staining, again before start of topical treatments on day 15 and at the end of the study on day 26 (Figure 8A). Corneal fluorescein staining was quantified by determining the fluorescence intensity of fluorescein on the cornea. Empty PLGA NP did not significantly affect corneal fluorescein staining (*p* = 0.21; Figure 8B). In contrast, xanthohumol-encapsulating PLGA NP (*p* < 0.05) and cyclosporine (*p* < 0.01) treatment resulted in a statistically significant reduction of corneal fluorescein staining.

### 3.5. Topically-Delivered Xanthohumol-Encapsulating PLGA NP Reduce Oxidative DNA Damage in Corneal Epithelial Cells In Vivo after Induction of Dry Eye Disease by Desiccating Stress/Scopolamine

**Figure 9:**
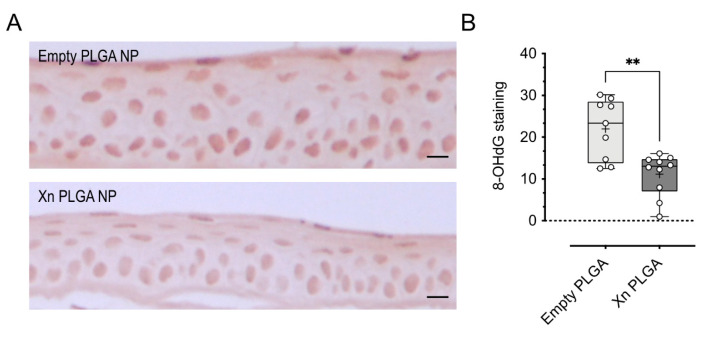
Xanthohumol-encapsulating PLGA NP reduce 8-OHdG immunoreactivity in corneal epithelial cells. (**A**) Representative examples of 8-OHdG immunoreactivity in corneal epithelial cells in empty and xanthohumol-encapsulating PLGA NP-treated eyes. (**B**) Quantification of nuclear intensity of staining revealed a statistically significant reduction of 8-OHdG staining by xanthohumol-encapsulating PLGA NP compared to empty PLGA NP. Data were analyzed by unpaired *t*-test and are shown as box and whisker plot, indicating the median (line), with the box extending from the 25th to 75th percentiles. Whiskers represent the range, while filled circles are individual data points from a single eye (*n* = 9–10 per group); the mean is indicated by a plus (+) sign. ** *p* < 0.01. Scale bar: 10 µM. Xn = xanthohumol.

In previous studies, we have shown that the desiccating stress/scopolamine model results in a significant amount of oxidative DNA damage that can be prevented by antioxidant treatment [8]. In order to determine the efficacy of xanthohumol-encapsulating PLGA NP, we stained corneal sections for 8-hydroxy-2′ deoxyguanosine (8-OHdG) and quantified immunoreactivity in corneal epithelial cells. Empty PLGA NP-treated eyes showed significant nuclear 8-OHdG immunoreactivity, as quantified by nuclear staining intensity; in contrast, xanthohumol-encapsulating PLGA NP showed a visible reduction in 8-OhdG staining intensity (Figure 9A). Quantification revealed a statistically significant reduction in 8-OHdG staining by 49.3 ± 7.3% (n = 9–10 per group; *p* < 0.01; Figure 9B).

This marked reduction in oxidative stress-associated DNA damage in corneal epithelial cells was not associated with marked changes in the histopathological properties of the cornea (Table 2). Specifically, epithelial and stromal thickness were not significantly affected by xanthohumol-encapsulating PLGA NP treatment.

## 4. Discussion

Our data provide strong in vitro and in vivo evidence that the natural compound, xanthohumol, can exert cytoprotective and antioxidative effects in preclinical models for dry eye disease. Specifically, xanthohumol and xanthohumol-encapsulating PLGA NP were cytoprotective against oxidative stress injury in human corneal epithelial cells. Furthermore, xanthohumol-encapsulating PLGA NP delivered topically reduced severity of corneal fluorescein staining and 8-OHdG labeling in the cornea, suggestive of reduced corneal damage and corneal oxidative DNA damage, respectively.

Previous studies have implicated increased cellular levels of oxidative stress in ocular surface disease. For example, lacrimal gland dysfunction can cause hyperosmolarity of the tear film [24], eliciting the generation of oxidative stress in human corneal epithelial cells [7]. Reactive oxygen species can activate nuclear factor-κB (NF-κB) [25], which regulates the endogenous antioxidant system, but also pro-inflammatory signaling through toll-like receptor 4 [26]. In our previous studies, we have shown that exposure to the desiccating stress/scopolamine model for dry eye disease causes significant increases in oxidative stress-mediated corneal damage [8], extending previous reports of apoptosis and damage to the corneal epithelium [25]. Therefore, the desiccating stress/scopolamine model for dry eye disease is a useful model to investigate the effects of antioxidants and antioxidant formulations on the ocular surface.

We used HCE-T cells as in vitro model to determine toxicity and efficacy of xanthohumol and xanthohumol-encapsulating PLGA NP (Figure 1, Figure 2, Figure 3, Figure 5 and Figure 6). While HCE-T cells are widely used, especially as they form a stratified epithelium with barrier properties and a characteristic morphology ([27]; for review, see [28]), HCE-T cells also display genomic abnormalities suggestive of some genetic drift [29], which must considered when interpreting in vitro findings derived from this cell line. 

Our mouse model for dry eye disease is based on a well-established paradigm that employs low-humidity air flow and concurrent scopolamine administration to induce dry eye disease in wild-type mice [30]. We have previously refined the model and its quantitative readouts used to assess dry eye disease severity for determining the efficacy of novel anti-dry eye disease therapeutics, including antioxidants [8]. The magnitude of changes, as well as the response of the positive control, ophthalmic cyclosporine (Restasis), were similar to those previously reported for this model [8,22,30].

Exposure to the desiccating stress environment with concomitant scopolamine administration resulted in a statistically significant reduction of tear volumes (~60%), showing successful induction of ocular surface disease (Figure 7A). In this study, all groups showed a statistically significant increase in tear volumes at the end of the 10-day treatment period, however, no statistically significant differences between vehicle, xanthohumol and cyclosporine-treated eyes were observed (Figure 7B). This suggests that the increase is primarily caused by lubrication of the cornea and tissues of the ocular surface, rather than due to a direct pharmacological effect. Here it may be important to note that tear volume measurements from mice are notoriously challenging and are easily confounded by physiological and environmental factors. 

To determine the pharmacological efficacy of xanthohumol, we used a PLGA NP-based formulation. PLGA NP are well-tolerated, biodegradable and approved by The United States Food and Drug Administration. 

The relationship between drug entrapment and NP size is complex, as reviewed by Astete and Sabliov [31]. The presence of drug can, therefore, result in reduced size, increased size, or unchanged size of PLGA NP. The use of polyvinyl alcohol as surfactant tends to result in consistent sizing of empty and drug loaded PLGA NP [32]. In accordance with others [33,34], we observed no significant change in nanoparticle morphology and size after drug encapsulation, as determined by electron microscopy and dynamic light scattering (Figure 4).

Release from PLGA NP occurs as NP degrade and is governed, in part, by the ratio of poly-lactic and poly-glycolic acid [35]. For this first proof-of-concept study, we used a ratio of 85:15 (poly-lactic:poly-glycolic acid), based on previously reported predicted release properties for the NP [21,36]. These properties were indirectly confirmed by determining the toxicity of xanthohumol-encapsulating PLGA NP in HCE-T cell cultures (Figure 5). In contrast to free xanthohumol, the nanoparticle formulation resulted in incomplete cell death at higher concentrations. This finding is expected when considering that xanthohumol release from PLGA NP occurs as HCE-T cultures continue to proliferate. Opposing effects of cytotoxic *versus* proliferative mechanisms are one of the commonly-recognized limitations of a monolayer culture system for cell lines. 

Similarly, the observed cytoprotective effect of xanthohumol-encapsulating PLGA NP against chemically-induced oxidative stress in vitro (Figure 6) is likely underestimated due to the limitations of the experimental model system, highlighting the importance of the in vivo proof-of-concept studies described herein.

One shortcoming of the current study is that PLGA (85:15) NP are negatively charged (Table 1). It is generally assumed that cationic NP exhibit enhanced retention times on negatively charged ocular tissues, such as the cornea and the conjunctiva [35,37]. Therefore, we opted to administer xanthohumol-encapsulating PLGA NP twice daily, matching the instillation frequency of cyclosporine. A detailed quantitative analysis of retention times of xanthohumol-encapsulating PLGA NP on the ocular surface is beyond the scope of this article, which to our knowledge provides the first preclinical proof-of-concept supporting the use of xanthohumol for ocular surface disease. Future studies will address modifications to PLGA NP formulations to include co-polymers such as chitosan or Eudragit RL100. For example, the latter, a copolymer of ethyl acrylate, methyl methacrylate and a low content of methacrylic acid ester with quaternary ammonium groups, has been successfully used for encapsulating cyclosporine with enhanced properties for topical delivery [38]. Nonetheless, xanthohumol-encapsulating PLGA NP showed similar efficacy when compared against 0.05% ophthalmic cyclosporine emulsion (Restasis; Figure 8), which is the current standard of care for patients with moderate to severe dry eye disease in the United States [2,39]. This finding demonstrates the potential for PLGA NP as a drug delivery vehicle for diseases of the ocular surface. Additional work characterizing xanthohumol-encapsulating PLGA NP, including release kinetics and stability is currently underway and beyond the scope of this initial demonstration of in vitro and in vivo efficacy.

Drug uptake into ocular tissues and specifically corneal epithelial cells depends critically on possible inhibitory effects on drug efflux transporters. P-glycoprotein 1 (P-gp) is an adenosine triphosphate-driven efflux pump, expressed in HCE-T cells and potently blocked by cyclosporine. To determine whether xanthohumol exerts inhibitory effects on P-gp, we performed an in vitro drug efflux transporter assay. No inhibitory effects of xanthohumol on P-gp were identified, while cyclosporine exhibited a typical dose response curve (Supplementary Material Figure S1). Caution is warranted when devising co-administration paradigms of xanthohumol with cyclosporine or other P-gp antagonists in order to avoid cytotoxic levels of xanthohumol. 

Xanthohumol is generally considered to exert its cytoprotective effects through both stimulating the dissociation of Keap1 from Nrf2 and direct ROS scavenging activity [14,15]. Typically, scavenging of ROS results in the diminishing activation of the phase II antioxidant system [40], reducing the endogenous antioxidant potential as cellular levels of oxidative stress fall. Given the potent activation of Nrf2 in HCE-T cells in the absence of oxidative stress (Figure 3), xanthohumol may be particularly well-suited for encapsulation in PLGA NP. In a previous study, we quantified the efficacy of three-times daily administration of the potent superoxide dismutase mimetic, manganese(III) tetrakis(1-methyl-4-pyridyl) porphyrin (Mn-TM-2-PyP). Intriguingly, xanthohumol-encapsulating PLGA NP had a much larger effect on 8-OHdG labeling in the cornea, reducing density of immunolabel by ~50% (Figure 9), compared with an ~25% reduction elicited by Mn-TM-2-PyP [8]. Given that the antioxidant potential of Mn-TM-2-PyP is significantly greater than that of xanthohumol ([8,17]), this finding may suggest that xanthohumol-encapsulating PLGA NP are not only able to be retained at the ocular surface for a prolonged period of time despite their negative surface charge, but also achieve sustained activation of the endogenous antioxidant system. 

## 5. Conclusions

Xanthohumol was cytoprotective against oxidative stress injury in human corneal epithelial cells, while xanthohumol-encapsulating PLGA NP significantly improved dry eye disease pathology in the mouse desiccating stress/scopolamine model. PLGA NP represent a safe and efficacious drug delivery vehicle for hydrophobic small molecules to the ocular surface. Future studies will optimize xanthohumol NP-based formulations with the goal to minimize instillation frequency, increase stability, and enhance efficacy.

## Figures and Tables

**Table 1:** Properties of PLGA NP formulations.

Parameter	Empty PLGA NP	Xn PLGA NP
Size (nm)	201.9 ± 0.1	191.0 ± 0.8
Polydispersity Index (PDI)	0.045 ± 0.009	0.029 ± 0.007
Zeta (ζ) potential (mV)	−21.6 ± 0.3	−24.8 ± 0.2
Encapsulation efficiency	N/A	13.1 ± 0.06%

**Table 2:** Histopathological properties of corneal tissue.

Parameter	Empty PLGA NP	Xn PLGA NP	Statistics
Thickness, corneal epithelium (µm) *	35.5 ± 3.3	30.8 ± 2.2	*n* = 10, *p* = 0.24
Thickness, corneal stroma (µm) *	178.9 ± 12.9	159.4 ± 7.8	*n* = 10, *p* = 0.21
Number of epithelial cell layers **	5 (4; 5.5)	4 (4; 5)	*n* = 10, *p* = 0.24

* Data are shown as mean ± SEM, or ** median (25th percentile; 75th percentile).

## Data Availability

The data presented in this study are available on request from the corresponding author (skaja@luc.edu).

## Supplementary Materials

The following are available online at https://www.mdpi.com/article/10.3390/pharmaceutics13091362/s1, Figure S1: Xanthohumol does not inhibit the drug efflux transporter, P-gp.

## Abbreviations

8-OHdG	8-hydroxy-2’-deoxyguanosine
ANOVA	One-Way Analysis of Variance
GAPHDH	glyceraldehyde 3-phosphate dehydrogenase
Keap1	Kelch-like ECH-associated protein 1
LDH	lactate dehydrogenase
MTT	3-(4,5-dimethylthiazol-2-yl)-2,5-diphenyltetrazolium bromide
Mn-TM-2-PyP	manganese(III) tetrakis(1-methyl-4- pyridyl) porphyrin
NF-kB	nuclear factor kappa B
NP	nanoparticle
Nrf2	nuclear factor erythroid 2-related factor 2
P-gp	P-glycoprotein 1
PBS	phosphate-buffered saline
PBS-T	phosphate-buffered saline supplemented with 0.2% *v/v* Tween-20
PLGA	poly(lactic-co-glycolic acid)
ROS	Reactive oxygen species
*t*BHP	*tert*-butyl hydroperoxide
TEM	transmission electron microscopy
Xn	xanthohumol
