# Supplementary material for: Poly(lactic-co-glycolic acid) Nanoparticles Encapsulating the Prenylated Flavonoid, Xanthohumol, Protect Corneal Epithelial Cells from Dry Eye Disease-Associated Oxidative Stress
Source: Pharmaceutics. 2021 Aug 30;13(9):1362. doi: 10.3390/pharmaceutics13091362 (PMC8471707; doi:10.3390/pharmaceutics13091362)
Supplement: Supplementary file 1 [file pharmaceutics-13-01362-s001.zip › pharmaceutics-1230098-supplementary.pdf]

pharmaceutics

MDPI

Supplementary Materials: Poly(Lactic-Co-Glycolic Acid) Nanoparticles Encapsulating the Prenylated Flavonoid, Xanthohumol, Protect Corneal Epithelial Cells from Dry Eye Disease-Associated Oxidative Stress

Anita Kirti Ghosh, Rubina Thapa, Harsh Nilesh Hariani, Michael Volyanyuk, Sean David Ogle, Karoline Anne Orloff, Samatha Ankireddy, Karen Lai, Agnė Žiniauskaitė, Evan Benjamin Stubbs Jr., Giedrius Kalesnykas, Jenni Johanna Hakkarainen, Kelly Ann Langert and Simon Kaja

Xanthohumol does not inhibit the drug efflux transporter, P-glycoprotein 1

Drug uptake into corneal epithelial cells and tissue levels depend on among other drug efflux transporters. With particular relevance to dry eye disease, cyclosporine is a potent inhibitor of P-glycoprotein 1 (P-gp), which is highly expressed in HCE-T cells. To determine whether xanthohumol exerts inhibitory effects on P-gp, we performed an *in vitro* drug efflux transporter assay.

To this end, HCET-T cells seeded in 96-well plates were exposed to a dose-range of either xanthohumol (10 nM – 10 μM) or to cyclosporine A (5 nM – 100 μM) for 30 min. Calcein-AM (2 μM) was added and fluorescence (excitation λ = 495 nm; emission λ = 525 nm) was measured in a Cytation 5 plate reader (BioTek Instruments, Inc.; Winooski, VT, USA) every 5 min for a 30 min period. The slope of the response was calculated and plotted over the drug concentration. Data were fitted using a Hill equation in Prism 9.0 (GraphPad, Inc., La Jolla, CA, USA).

Xanthohumol had no effect on P-gp drug transporter function. The slope did not change with an increasing concentration of Xanthohumol (Suppl. Fig. 1) suggesting the absence of an inhibitory effect on P-gp. In contrast, cyclosporine resulted in a dose-dependent increase in the slope that could be fitted with a Hill equation, confirming the inhibitory effect of cyclosporine on P-gp (supplementary material figure S1).

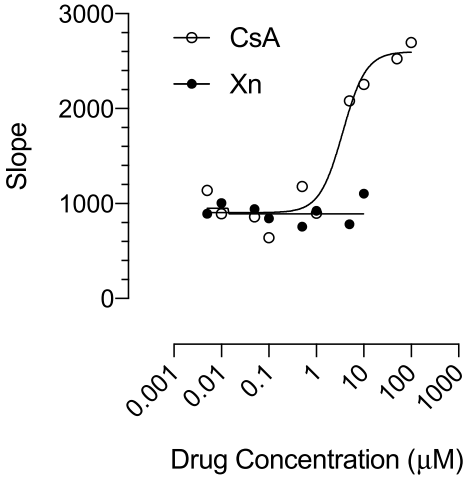

Figure S1. Xanthohumol had no effect on P-gp drug transporter function. Xanthohumol (filled circles) had no effect on the slope, while cyclosporine (open circles) showed a dose-dependent increase that was fitted using a Hill equation. Data were calculated from 8 technical replicates per concentration and are presented as mean.

Given the potent effects of cyclosporine on P-gp, care should be taken when co-administering ocular topical drugs together with cyclosporine, as this may results in increased concentrations in the corneal epithelial cells due to inhibition of P-gp.
